# Racialized Sex-Based Harassment: A U.S.-Based Intersectional Framework for Understanding Harassment of Black Women and Men

**DOI:** 10.3390/bs16020184

**Published:** 2026-01-27

**Authors:** Darius M. Washington, Tuyen K. Dinh, Margaret S. Stockdale

**Affiliations:** Department of Psychology, Indiana University—Indianapolis, 402 N. Blackford St., Indianapolis, IN 46202, USA; tkdinh@iu.edu (T.K.D.); pstockda@iu.edu (M.S.S.)

**Keywords:** sexual harassment, sex-based harassment, racialized sex-based harassment, stereotypes, intersectionality, intergroup threat, social dominance orientation

## Abstract

Although scholarship has long called for attention to the intersection of race and gender in workplace harassment, the experiences of Black Americans remain insufficiently theorized. Existing frameworks often assume harassment to be gender-based in ways that center White women’s victimization, leaving limited conceptual space to understand how Black women and Black men are targeted. In this essay, we synthesize research on racialized sex-based harassment (RSBH) to illustrate how harassment directed at Black Americans is shaped by cultural narratives that simultaneously sexualize, criminalize, and devalue them. Specifically, we introduce sociohistorical archetypes (e.g., Jezebel, Mammy, Sapphire, Mandingo, Brute, Uncle Tom) as cultural mechanisms through which RSBH is enacted, rationalized, and normalized within organizational contexts. We argue that RSBH functions as a mechanism for enforcing racialized gender hierarchy: it draws on sociohistorical meanings attached to Black femininity and masculinity to mark certain identities as inherently available, threatening, or subordinate. We further review evidence linking RSBH to psychological distress, social identity threat, physiological strain, and career stagnation, as well as factors that shape vulnerability and adaptation. By conceptualizing RSBH as a patterned and predictable form of identity-based harm, grounded in the lasting impact of sociohistorical archetypes, rather than a variation of generalized sexual harassment, this work advances theories of harassment and race in organizations. We conclude by outlining implications for measurement, organizational policy, and intervention efforts aimed at disrupting the reproduction of racialized gender inequality at work.

## 1. Racialized Sex-Based Harassment: A Critical Gap in Harassment Theory and Measurement

Despite decades of research documenting sexual harassment, much of this work has centered on the experiences of White women. While this focus has been invaluable, it has overshadowed the distinct and pervasive harassment targeting racial minority groups—particularly Black Americans—in U.S. workplaces. In male-dominated fields and highly hierarchical institutions like the military, racial minority employees often face greater risks of harassment, which prior research has documented and that we extend by naming this “racialized sex-based harassment” (RSBH; Buchanan & Ormerod, 2002; Settles et al., 2012). These experiences carry profound consequences for psychological and physical health (Buchanan et al., 2007, 2008; Cortina & Berdahl, 2008; Fitzgerald et al., 1999; Quick & McFadyen, 2017; Raj et al., 2021), including diminished job satisfaction, increased absenteeism, stalled career advancement, and reputational harm (Holland & Cortina, 2016; McLaughlin et al., 2017; Solomon & Williams, 1997). This conceptualization is situated within the sociohistorical context of the United States, where racialized gender hierarchies have been produced through slavery, segregation, and labor stratification and continue to shape organizational intra and intergroup relations. Although harassment occurs globally, the archetypes and mechanisms examined here reflect distinctly U.S. based racial tropes that inform how Black women’s and Black men’s identities are interpreted and regulated at work.

Although recent U.S. Equal Employment Opportunity Commission (EEOC) data would suggest harassment toward Black Americans warrants closer investigation, limited progress has been made toward theorizing the mechanisms through which these victims experience harassment. Statistics demonstrate a surge in race-based sexual harassment charges: 8524 in Fiscal Year 2022, 11,720 in 2023, and 12,863 in 2024 (EEOC, n.d.). Yet, existing harassment frameworks and measurements fail to adequately depict how harassment itself functions as a tool through which gendered racism is enforced. This recognition underscores a critical gap: most frameworks and extant standardized measures, including the Likelihood to Sexually Harass scale (Pryor, 1987), the Sexual Experiences Questionnaire (SEQ; Fitzgerald et al., 1988, 1995), the Workplace Crush Scenario (Williams et al., 2017), and the Sex-Based Harassment Inventory (SBHI; Grabowski et al., 2022), which remain grounded in assumptions that either center White women’s victimization or fail to account for the racialized and gendered meaning(s) that shape how harassment is enacted toward racial minority victims. Specifically, these measures do not capture harassment rooted in sociohistorical archetypes that uniquely target Black individuals, such as hypersexualization (e.g., Jezebel, Mandingo; Bogle, 2016; West, 1995), threat (e.g., Sapphire, Brute, Angry Black Man; Bogle, 2016; Pilgrim, 2000a), and subordination (e.g., Mammy, Uncle Tom) (Berdahl & Moore, 2006; Buchanan, 2016; West, 1995). When these realities are excluded from measurement, RSBH remains underassessed, perpetuating institutional blind spots.

Social movements like #MeToo and #TimesUp have amplified awareness of sexual harassment, but their narratives have disproportionately centered on White women, leaving racial minority women and men at the margins (Leung & Williams, 2019). Black women are often perceived as non-prototypical victims, undermining their credibility and support (Davis, 2023; Purdie-Vaughns & Eibach, 2008). Similarly, Black men face dual invisibility: cultural myths about masculinity (e.g., hypersexuality, aggression, and resilience) and race render them unlikely victims in the public imagination (Cesario, 2020; Curry, 2019), reinforcing stereotypes and limiting empathy and organizational accountability. The crux of the matter is that these blind spots not only distort scientific understanding but directly contribute to ongoing institutional failures and policy gaps.

The present paper addresses these gaps by introducing RSBH as a distinct phenomenon and proposing an integrative framework to explain its underlying mechanisms. Drawing on intersectionality theory (Crenshaw, 1989), social dominance theory (Sidanius & Pratto, 1999), integrated threat theory (Stephan & Stephan, 2000), and masculinity threat (Vandello et al., 2008), we argue that RSBH operates as a tool for enforcing racialized gender hierarchies. Specifically, we examine how sociohistorical stereotypes and perceived intergroup threats converge to motivate harassment and how these dynamics produce unique psychological, behavioral, and organizational consequences (see Cortina & Areguin, 2021). By advancing a novel framework specific to the ways in which racial minorities experience harassment, we aim to expand current models of harassment and respond to calls for models that capture intersectional dynamics of race and gender that shape experiences of harassment. Practical implications for organizational intervention and policy development are discussed, emphasizing the urgent need to recognize and disrupt these layered forms of harm.

## 2. Overview of Conceptual and Theoretical Framework

RSBH cannot be fully accounted for by existing models of sexual harassment or sex-based harassment. Sexual harassment frameworks primarily conceptualize harassment in terms of sexualized conduct and coercion (Fitzgerald et al., 1995), whereas sex-based harassment emphasizes the policing of gender norms and the protection of status hierarchies (Berdahl, 2007a; Berdahl & Moore, 2006; Cortina & Berdahl, 2008). In both approaches, race is typically treated as a contextual variable rather than a constitutive feature of how harassment is enacted and interpreted. We conceptualize RSBH as an extension of these frameworks that centers the racialization of gender, demonstrating how sociohistorical meanings attached to Black femininity and masculinity systematically shape the targets, content, and consequences of harassment in organizational settings.

**Figure 1:**
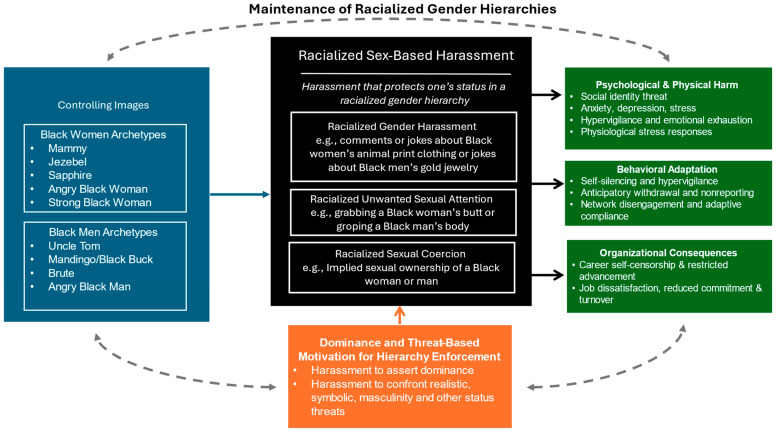
Conceptual Model of Racialized Sex-Based Harassment Dynamics.

Figure 1 presents an integrative conceptual model illustrating how RSBH extends sexual harassment and sex-based harassment by incorporating the racialization of gender as a core mechanism. The model shows how behaviors commonly categorized as gender harassment, unwanted sexual attention, or sexual coercion take on distinct meanings when enacted within racialized gender hierarchies, operating as mechanisms of racialized discipline rather than isolated interpersonal acts. Viewed in this way, RSBH is not a peripheral variant of existing constructs but a patterned strategy through which intersecting systems of race and gender hierarchy are maintained.

### 2.1. Psychological Definitions of Sexual Harassment

Sexual harassment has traditionally been defined as unwelcome sexual advances, requests for sexual favors, and other verbal or physical conduct of a sexual nature that creates a hostile or offensive work environment (EEOC, n.d.; Fitzgerald et al., 1995). In workplace research, sexual harassment is commonly conceptualized as comprising three distinct but related dimensions: gender harassment, unwanted sexual attention, and sexual coercion (Fitzgerald et al., 1988, 1995; Gelfand et al., 1995).

Gender harassment refers to verbal or nonverbal actions that convey insulting, demeaning, or degrading attitudes about men and women at work (Fitzgerald et al., 1995; see also Leskinen & Cortina, 2014; Leskinen et al., 2011). Within this category, two types exist: sexual and sexist hostility. Both forms of harassment center around disparagement based on gender, and we theorize that race also plays a crucial role. Examples of gender harassment include sexist joking, displaying pornographic content, or making obscene gestures. For men, it often centers on punishing those who fail to meet masculine standards and pressuring them to conform to these ideals (N. M. Alonso et al., 2025; Berdahl & Moore, 2006; Stockdale et al., 1999; Waldo et al., 1998). This form of harassment often referred to as “gender-role harassment” is the most prevalent form among men and is frequently used as a tool for policing male gender norms (Berdahl et al., 1996; Cortina & Areguin, 2021).

Unwanted sexual attention pertains to expressions of sexual interest that are unwelcome and unrequited, including repeated requests for dates, nonconsensual touching, or sexual assault (Fitzgerald & Cortina, 2018). These behaviors are characterized by their persistence and lack of reciprocation and can be perpetrated by individuals of any gender (Fitzgerald et al., 1995; Kabat-Farr & Cortina, 2014; Waldo et al., 1998). Lastly, sexual coercion involves the promise of rewards or the threat of punishment in exchange for sexual favors at work (Fitzgerald & Cortina, 2018). This may include threatening demotion unless sexual acts are performed, or by offering promotions or other benefits contingent upon sexual compliance (Fitzgerald et al., 1995; Stockdale et al., 1999).

These three dimensions are commonly assessed using the Sexual Experiences Questionnaire (SEQ; Fitzgerald et al., 1995), a tool originally developed from the experiences of White women and primarily focused on women’s experiences. While the SEQ has since been adapted to capture the experiences of male targets and other groups (see Cortina, 2001; Waldo et al., 1998), its original construction did not account for intersectional identities or culturally specific forms of harassment faced by men, Black men, and Black women (Berdahl & Moore, 2006; Buchanan, 2016).

### 2.2. Sex-Based Harassment

Equally important, the definition of harassment has evolved as scholars recognize harassment is not limited to overt sexual advances but also includes behaviors that derogate, demean, or humiliate individuals based on their sex or gender (Berdahl, 2007a). Berdahl (2007a) advanced the concept of “sex-based harassment,” broadening the definition to encompass any behavior that enforces or polices gender norms, regardless of the victim’s gender. This innovation reframes harassment as a mechanism for protecting social status and maintaining gender hierarchy, rather than simply expressing sexual desire (N. M. Alonso et al., 2025; Berdahl, 2007a; Stockdale et al., 1999).

For Black Americans, harassment is rarely experienced as solely sex-based or solely race-based. Instead, race and gender operate simultaneously to produce forms of mistreatment that are fundamentally different in content and consequence (Buchanan, 2016; Crenshaw, 1989). Black women are not only subordinated as women, but are hypersexualized, criminalized, and rendered emotionally deviant in ways that make them especially vulnerable to both sexualized coercion and credibility threats when they resist harassment (Buchanan & Fitzgerald, 2008; Collins, 2004; West, 1995). Likewise, Black men are assumed to embody hypermasculinity and sexual danger and therefore become targets of both emasculating harassment and racialized sexual suspicion designed to maintain the racial–gender status quo (Bogle, 2016; Pilgrim, 2000a). These realities require a theoretical shift from additive models of discrimination to an understanding of RSBH as a distinct and patterned form of harm.

### 2.3. Racialized Sex-Based Harassment

Building on this expanded framework, RSBH reflects a form of identity-based mistreatment that is rooted in the racialization of gender and the gendering of race. Specifically, RSBH refers to harassment that demeans, sexualizes, or humiliates Black individuals through stereotypes that simultaneously mark them as subordinate and threatening in both racial and gender hierarchies (see Buchanan & Ormerod, 2002; West, 1995). This formulation departs from traditional sexual harassment scholarship, which has conceptualized harassment primarily as a tool to enforce gender hierarchy (Berdahl, 2007a), and from racial harassment frameworks focused on ethnic hostility and exclusion (Cortina et al., 2013). For Black Americans, harassment reflects the interlocking nature of racial and gender systems of oppression, such that neither axis alone sufficiently explains the content or motives of these experiences (Crenshaw, 1989). RSBH functions as a tool for enforcing racialized gender hierarchies and protecting hegemonic status (see N. M. Alonso et al., 2025; Berdahl, 2007a), but it is not limited to majority group members (e.g., White men). Individuals across social groups, including other marginalized groups and even in-group members, may engage in these behaviors because stereotypes, cultural archetypes, and societal narratives that sustain racialized gender hierarchies permeate broadly and shape norms of dominance and subordination. These dynamics underscore that harassment is not simply interpersonal misconduct but a mechanism for maintaining power structures (N. M. Alonso et al., 2025; McLaughlin et al., 2012; Uggen & Blackstone, 2004), often activated when identities or roles are perceived as threatening to the gendered racial order.

The content of RSBH is anchored in racialized archetypes that emerged from slavery and have been continually reproduced through media, policy, and organizational practices (Collins, 2004; Pilgrim, 2000a, 2000b). For Black women, these include tropes of hypersexuality, servility, and anger—characteristics perpetrators draw upon to justify harassment and deny victimhood (Buchanan, 2016; West, 1995). For Black men, harassment is often grounded in myths of sexual aggression, criminality, dominance, and subservience, creating a paradox whereby they are simultaneously punished for embodying masculinity either too much or too little (Bogle, 2016; Pilgrim, 2000a). These patterns place Black individuals in a double bind: any behavior—assertiveness, ambition, refusal of sexual advances, emotional expression—can be interpreted as a violation warranting discipline (Funk & Werhun, 2011). Because racialized gender stereotypes differ from those applied to White targets, harassment toward Black Americans cannot be understood as extensions of generalized sexism. Whereas women who violate feminine norms may be harassed for threatening male status (Berdahl, 2007b; Grabowski et al., 2022), Black women are penalized even when fulfilling feminine-coded roles due to assumptions of inherent deviance (Hall et al., 2015). Similarly, while gender-role harassment toward men often seeks to feminize or humiliate, Black men are frequently targeted for being too masculine—too confident, too strong, too sexually capable—which is interpreted as threatening White men’s dominance and White women’s purity (Ferber, 2007; Hester & Gray, 2018; hooks, 2004; Johnson, 2018; Staples, 1978). These deviations activate racialized gender threats that extend far beyond conventional gender harassment models.

Despite compelling qualitative evidence documenting these patterns (Berdahl, 2007b; Buchanan, 2016; Buchanan & West, 2010; Collins, 2004), current harassment measures do not adequately capture the racialized sexualized content of harassment experienced by Black workers (National Academies of Sciences, Engineering, and Medicine, 2018). When measurement tools fail to detect these grievances, organizational systems ignore them, reinforcing cultures that tolerate and reproduce harm (see Holland & Cortina, 2016). Although the existing measurement literature has made important strides toward recognizing intersectional forms of harassment, this work has not always been situated within an explicit theoretical account of racialized gender policing and status maintenance. An important contribution in this area is the Racialized Sexual Harassment Scale (RSHS) introduced by Buchanan (2016) in an unpublished technical report. The RSHS was developed to assess harassment experiences that occur at the intersection of race and gender and that are not adequately captured by traditional, gender-neutral measures of sexual harassment. The scale includes items reflecting differential treatment, sexualized scrutiny, and demeaning behavior toward Black women and Black men—forms of mistreatment that are consistent with, though not explicitly labeled as, racialized gender stereotypes documented in the broader literature.

Importantly, while the RSHS items themselves do not directly invoke specific sociohistorical archetypes (e.g., Jezebel, Mammy, Mandingo, Angry Black Man/Woman), which we elaborate on below, Buchanan and her colleagues have elsewhere explicitly theorized the role of such controlling images in shaping the content and meaning of harassment directed at Black individuals (e.g., Buchanan & Ormerod, 2002; Buchanan & Fitzgerald, 2008; see also Collins, 2004; Szymanski & Lewis, 2016). Across this body of work, Buchanan’s scholarship has been instrumental in demonstrating that Black women and Black men experience harassment in ways that are qualitatively distinct from those captured by widely used measures such as the SEQ. For example, Buchanan’s Racialized Sexual Harassment Scale (RSHS) assesses whether targets experience harassment because of gender, ethnicity, or the intersection of both, and includes explicit examples demonstrating race–gender stereotyping and sexualized degradation (Buchanan, 2016). The scale’s item examples capture how racialized stereotypes are enacted interpersonally—e.g., statements that “Black women are rude,” racialized gendered slurs (e.g., “black bitch”), and sexualized body commentary tied directly to race (e.g., comments about a Black woman’s “Black ass” or a Black men’s large penis; Buchanan, 2016). These examples are useful here not because any single instrument resolves the conceptual problem, but because they illustrate the central claim of RSBH: harassment becomes qualitatively different when it is organized around racialized gender scripts.

### 2.4. Theoretical and Conceptual Framework

A comprehensive understanding of RSBH requires integrating multiple theoretical perspectives that illuminate how intersecting systems of power shape the lived experiences of Black Americans in social and workplace settings. Intersectionality theory (Crenshaw, 1989) provides a foundational lens, emphasizing that the convergence of race and gender produces unique forms of oppression that cannot be understood by examining either axis in isolation. This framework is essential for capturing the ways in which Black women and men experience harassment that is simultaneously racialized and gendered.

As aforementioned, RSBH does not occur randomly; it operates as a targeted response to perceived challenges against the racialized gender hierarchy and the social power structures embedded within it. Building on foundational accounts of harassment as dominance enforcement (Berdahl, 2007a; Franke, 1997), RSBH reflects an attempt to discipline Black individuals who threaten existing social order through their presence, competence, or refusal of subordination. This enforcement arises from both structural systems of inequality and individual psychological motives that intertwine to preserve power asymmetries between groups.

Sociological perspectives on power and discipline extend these explanations by clarifying how RSBH operates as a mechanism of regulation. From a Foucauldian perspective, power is exercised not only through formal authority or overt sanction, but through everyday organizational norms, practices, and forms of surveillance that define what behaviors are legitimate and which invite correction (Foucault, 1975/1977, 1976/1978). Within this view, harassment functions as a disciplinary signal that marks Black women’s and Black men’s behavior as “out of place,” thereby encouraging anticipatory self-regulation. As such, Black women and men are likely to adjust their behavior based on expected social or organizational sanctions, rather than in response to immediate punishment; power operates by making the consequences of deviation foreseeable, thereby encouraging self-monitoring and behavioral constraint (Foucault, 1975/1977). In the context of racialized sex-based harassment, repeated exposure to sexualized, demeaning, or credibility-undermining responses teaches Black employees which behaviors carry risk, fostering silence, constrained self-presentation, withdrawal, and nonreporting as rational adaptations to anticipated retaliation, disbelief, or social costs (Bergman et al., 2002; Hershcovis et al., 2021; McLaughlin et al., 2017). Organizational scholarship on sexual harassment has shown that such disciplinary dynamics foster self-monitoring, boundary policing, and behavioral constraint, even in the absence of ongoing overt mistreatment (Brewis, 2001; Brunner & Dever, 2014).

Social dominance theory posits that people differ in the extent to which they believe hierarchical group relations are justified, stable, and desirable (Sidanius & Pratto, 1999). Individuals high in social dominance orientation (SDO) are more supportive of discriminatory practices that maintain dominance, including sexual harassment (Kteily et al., 2012; Russell & Trigg, 2004). Because Black Americans are positioned at the intersection of both racial and gender subordination relative to White men, their successes or assertions of equal status evoke stronger hierarchy threats. Harassers respond by reasserting dominance through tactics that emphasize sexualized inferiority, aiming to restore group-based power advantages (Branscombe et al., 1999).

Integrated threat theory (ITT) further explains these reactions by distinguishing between realistic threats to access and symbolic threats to cultural supremacy (Stephan & Stephan, 2000). Stated differently, ITT explains prejudice and discrimination as responses to perceived threats from outgroups (Stephan & Stephan, 2000). The theory identifies two primary categories of threat: realistic threats, which involve concerns about tangible resources such as jobs, safety, or economic opportunities, and symbolic threats, which refer to perceived challenges to cultural values, norms, and identity. In professional settings, a Black woman who excels as a leader can be perceived as undermining male authority and transgressing normative expectations of femininity (Berdahl, 2007b; Berdahl & Moore, 2006). Professionally successful Black men may face backlash when their competence and ambition are perceived as threatening. Research demonstrates that Black men are more likely to be accepted in high-status roles when they appear warm or non-threatening, a pattern known as the “teddy-bear effect,” which reflects racialized assumptions that position Black masculinity as inherently threatening (Livingston & Pearce, 2009). RSBH functions as a corrective response: sexualized and dehumanizing comments, coercive attention, humiliation, and intimidation are used to put racial minority employees “back in their place” and delegitimize their authority in the eyes of others.

Importantly, power-based explanations of harassment offer additional insight into how perpetrators perceive and respond to perceived threats to the racial–gender hierarchy posed by Black targets. Power not only shapes who is positioned to harass, but also how harassment becomes a strategic tool to secure compliance and assert ownership over social space (C. Anderson & Berdahl, 2002). Furthermore, power does not merely constrain; it activates. Feelings of power increase approach motivation and appetitive impulses, heightening a readiness to act on desires—including those that demean, objectify, or subordinate others (Guinote, 2017; Keltner et al., 2003; Stockdale et al., 2019). When power is coupled with longstanding racialized and sexualized stereotypes, Black men and women become especially vulnerable targets: power magnifies the salience of these archetypes and increases the likelihood that those in dominant-group positions will treat Black individuals as available, usable, or controllable (Gruenfeld et al., 2008; Stockdale et al., 2019).

In this way, harassment toward Black women often reflects entitlement to their bodies, emotional labor, and sexual accessibility (see). Harassment toward Black men, by contrast, functions to regulate and contain their masculinity, physical presence, and perceived threat (Bogle, 2016). Both outcomes reaffirm a shared expectation that Black workers must remain accessible yet nonthreatening, competent yet deferential, and visible yet silent about their mistreatment. Harassment thus operates not merely as interpersonal misconduct but as a mechanism of racialized gender governance—one that polices the boundaries of who may claim space, autonomy, or dignity in organizational (and social) life.

Masculinity threat processes also contribute to harassment toward Black men, though the dynamics differ markedly from those involving White men. Cultural narratives frame Black masculinity as simultaneously overdeveloped and inferior: excessively physical, sexually aggressive, and emotionally volatile (Collins, 2004; Pilgrim, 2000a). When Black men embody professional competence or interpersonal confidence, perpetrators may experience symbolic emasculation, leading them to deploy harassment to reestablish dominance and undermine Black men’s masculinity. Conversely, when Black men display gentleness, fear, or vulnerability—behaviors cast as nonnormative—they are targeted in ways that belittle and feminize them. These contradictory expectations reveal that Black men’s masculinity is hyper-scrutinized and perpetually penalized, regardless of behavior. RSBH may be especially prevalent in “masculinity contest cultures,” where attributes like toughness and competitiveness are valorized (N. Alonso, 2018; N. M. Alonso et al., 2025; Berdahl et al., 2018; Glick et al., 2018), further marginalizing those who do not conform or identify with traditional masculinity ideals (e.g., LGBTQ+ individuals).

Finally, RSBH is exacerbated by cultural narratives that deny Black people vulnerability and legitimate suffering. Black women are assumed to be servile and promiscuous; Black men are assumed to be aggressive and threatening. These beliefs reduce empathy toward Black American targets, silence among victims, and lower organizational willingness to intervene (Hershcovis et al., 2021). As a result, perpetrators may feel emboldened to police Black identity through harassment with little fear of accountability or social sanction. In sum, RSBH functions as a systemic strategy to keep Black individuals “in their place”—a mechanism that expresses and reproduces supremacy under the guise of interpersonal conflict (see Stephan & Stephan, 2000).

## 3. Archetypes of Black Women and Black Men

The lived RSBH experiences of Black women and Black men are deeply shaped by enduring cultural archetypes—symbolic figures that originated in the era of slavery and have been perpetuated through social, legal, and U.S. organizational structures into the present day (Bogle, 2016; Buchanan, 2016; Collins, 2004; hooks, 2004; Pilgrim, 2000a, 2000b). These archetypes evoke racialized stereotypes of criminality, hypersexualization, subservience, and dehumanization. In organizational life, such stereotypes are not remnants of a distant past but active interpretive frames that shape how colleagues and supervisors perceive and judge Black employees’ behavior (see Hekman et al., 2017). When these stereotypes are triggered, harassment becomes a culturally sanctioned corrective response to perceived violations to the racialized gender hierarchy.

### 3.1. Mammy, Jezebel, and Sapphire

For Black women, three principal archetypes have historically defined their representation: the Mammy, Jezebel, and Sapphire. The Mammy figure depicted Black women as loyal, nurturing, and asexual caretakers, whose primary value lay in their service to others. This stereotype was instrumental in rationalizing the exploitation of Black women during slave labor while simultaneously denying their autonomy and sexuality (Collins, 2004; Cheeseborough et al., 2020; West, 1995). In contrast, the Jezebel trope casts Black women as hypersexual and promiscuous, a narrative that was used to legitimize sexual violence and objectification, positioning Black women as inherently available and lacking virtue (J. R. Anderson et al., 2018; Buchanan, 2016; Collins, 2004). As a result, sexual harassment such as unwanted comments, touching, or rape is rationalized as natural or consensual, undermining Black women’s claims to victimization (Buchanan, 2016). The Sapphire archetype, often referred to as the “Angry Black Woman,” emerged as a response to Black women’s resistance and assertiveness, framing them as hostile, emasculating, and deserving of exclusion or harsher discipline in both social and professional contexts (Buchanan & Ormerod, 2002; Collins, 2004; Lewis et al., 2016).

Additionally, controlling images such as the Strong Black Woman/Superwoman ideology suggest that Black women possess limitless resilience and do not experience harm like others, which reduces empathetic responses from coworkers and invalidates requests for organizational support (Donovan & West, 2015; Nelson et al., 2016; Woods-Giscombé, 2010). Finally, other emerging stereotypes include the Welfare Queen/Gold Digger stereotype, which depicts Black women as manipulative and financially opportunistic. This image constructs harassment as justified suspicion of deceit, or as necessary punishment to curb their presumed exploitation of resources or power (Collins, 2004; Hancock, 2004; Harris-Perry, 2011). Together, these intersecting stereotypes strip Black women of credibility, bodily autonomy, and organizational legitimacy, making them disproportionately vulnerable to harassment that serves to keep them subordinate.

### 3.2. Mandingo, Brute, Uncle Tom

Black men are similarly constrained by an interconnected set of racialized gender stereotypes that mark them as hypervisible, dangerous, and in need of control (Bogle, 2016, Collins, 2004; Pilgrim, 2000a; Settles et al., 2012). The Mandingo/Black Buck stereotype paints Black men as sexually dominant and insatiable, legitimizing disproportionate scrutiny of their sexual interactions and portraying harassment directed at them as necessary boundary monitoring (Pilgrim, 2000a). The Mandingo stereotype further criminalizes Black male sexuality by framing Black men as deviant and predatory threats to White women, thereby legitimizing coercive control in the workplace and enabling identity policing under the guise of “safety.” The Brute stereotype depicts Black men as inherently violent and unpredictable, such that expressions of confidence, ambition, or leadership are interpreted as threats requiring suppression (Goff et al., 2008; Johnson, 2018). Simultaneously, Black men are targeted by emasculation stereotypes, where emotional expression or perceived weakness is ridiculed to reinforce their lower status in the racial–gender hierarchy (Collins, 2004). The Uncle Tom stereotype reinforces expectations of docility and subservience; when Black men refuse subordinate roles, harassment escalates as punishment for perceived insubordination (Bogle, 2016; Collins, 2004). This paradox—being punished for both embodying and contradicting dominant masculinity scripts—places Black men under constant vigilance and threat, no matter their response.

These archetypes, summarized in Table 1, are not simply biased beliefs; they are cultural scripts that authorize harassment and oppression. They transform discriminatory actions into “reasonable responses” to identity violations or threats to one’s status. When a Black woman rejects inappropriate advances, she may be framed as frigid or hostile; when a Black man advocates for fairness, he may be labeled aggressive. Harassment is thus embedded in a logic of racialized gender policing. The workplace becomes a site of continuous surveillance and correction, enforcing who is allowed to belong, who must remain subordinate, and who is punished for deviating from roles that prop up White patriarchal power.

However, unlike for Black women, masculinity lies at the core of harassment experiences for men, as sexual harassment frequently operates as a mechanism for policing male gender norms and punishing those who fail to conform to dominant masculine standards (N. Alonso, 2018; N. M. Alonso et al., 2025; Berdahl, 2007b; Waldo et al., 1998). “Don’t be a girl and don’t be gay,” (see N. Alonso, 2018) encapsulates this sentiment, where non-conformity to traditional masculinity is deemed inferior. Boys and men who are effeminate (or perceived to be feminine) face this inferior status, often through “masculinity teasing,” a form of harassment intended as put-downs and a method of masculine socialization (Stockdale et al., 1999). Research by Vandello et al. (2008) on precarious manhood suggests that manhood status can be both earned and lost, offering insights into individual motivations for engaging in RSBH, reinforcing traditional racial-gender hierarchies in the process. For Black women and Black men, harassment is often compounded by intersecting racial and gender stereotypes, resulting in experiences that include (racialized) gender harassment, unwanted sexual attention, and sexual coercion—each shaped by controlling images such as the Jezebel, Mammy, Sapphire, Mandingo, Brute, Angry Black Woman/Man, and Uncle Tom tropes (Buchanan, 2016). These forms of harassment not only undermine psychological and occupational well-being but also reinforce social hierarchies and perpetuate exclusion within the workplace.

In sum, the archetypes of Black women and Black men are deeply embedded in the historical and contemporary fabric of American society. Their continued operation in workplaces contributes to the unique, intersectional vulnerabilities and experiences of RSBH among Black individuals. Addressing these patterns requires not only recognition of their historical roots but also targeted interventions that disrupt their reproduction in organizational culture and practice.

## 4. Behavioral Manifestations of RSBH

This historical background provides an opportunity to revisit Fitzgerald et al.’s (1995) tripartite definition of sexual harassment and Buchanan’s (2016) RSHS to account for racialized sex-based harassment, which we explore below.

### 4.1. Racialized Gender Harassment

Racialized gender harassment involves hostile, degrading, or exclusionary conduct that polices racialized gender norms and reinforces group-based hierarchies. In the RSHS, this process is reflected in items assessing insults and expectations targeting individuals specifically because of the intersection of their gender and ethnicity (Buchanan, 2016). For example, the scale includes harassment that casts Black women through racialized gender stereotypes (e.g., “Black women are rude”) and behavioral regulation that presumes racialized gender deviance (Buchanan, 2016). These behaviors map onto long-standing controlling images that depict Black women as hostile or irrational—stereotypes that legitimize punitive reactions when Black women assert competence, set boundaries, or refuse subordination (Buchanan & Ormerod, 2002; Lewis et al., 2016).

For Black men, racialized gender harassment often takes the form of masculinity policing under conditions of racialized threat. When Black men’s assertiveness is read as danger and their restraint as weakness, harassment functions to enforce a narrow band of “acceptable” racialized masculinity and to sanction deviations from it (Hammond, 2012; Settles et al., 2012). In this way, RSBH clarifies what traditional accounts sometimes leave implicit: gender harassment is not merely gendered hostility—it can operate as racialized discipline that regulates voice, ambition, visibility, and credibility across organizational settings (Berdahl, 2007a; Crenshaw, 1989).

### 4.2. Racialized Unwanted Sexual Attention

Racialized unwanted sexual attention encompasses unwelcome sexual comments, advances, or scrutiny that are imbued with racialized sexual meanings. Here, the defining feature is not the sexual content alone, but the way racial archetypes shape what the behavior communicates and what it permits others to do to the target. Several RSHS examples illustrate this mechanism directly, including sexualized body commentary that explicitly links race and gender (e.g., comments about a Black woman’s “Black ass” or assumptions about a Black man’s sexual endowment), as well as racialized gendered slurs (e.g., “black bitch”; Buchanan, 2016). These exemplars are consistent with a broader literature showing that Black women are disproportionately positioned through hypersexualized scripts (e.g., Jezebel), which increases sexual objectification and renders sexual intrusion more socially “available” to perpetrators (J. R. Anderson et al., 2018; Szymanski & Lewis, 2016).

Critically, scholarship on harassment among Black women indicates that sexualized attention is often intertwined with racialized meanings—particularly in cross-racial contexts—such that sexual advances and commentary are accompanied by assumptions of availability, deviance, or disposability (Berdahl & Moore, 2006; Woods-Giscombé, 2010). For Black men, sexualization can be organized through stereotypes of sexual excess or danger (e.g., Mandingo/Brute logics), producing a form of unwanted sexual attention that is simultaneously objectifying and threatening. RSBH contributes by making explicit that “unwanted sexual attention” is not a uniform phenomenon: it can function as racialized social control when it assigns targets to dehumanizing sexual scripts and constrains their ability to refuse, report, or be believed without incurring penalty (Crenshaw, 1989; Settles et al., 2012).

### 4.3. Racialized Sexual Coercion

Racialized sexual coercion refers to situations in which sexual compliance is implicitly or explicitly tied to material outcomes, safety, or organizational access under conditions of unequal power. Harassment frameworks recognize sexual coercion as a defining subtype of sexual harassment (Fitzgerald et al., 1995; National Academies of Sciences, Engineering, and Medicine, 2018), and organizational research demonstrates that coercion can operate through dependence, retaliation risk, and institutional credibility dynamics—not only through overt quid pro quo demands (Cortina & Magley, 2009; McLaughlin et al., 2012). While the RSHS does not directly measure explicit quid pro quo sexual coercion, it captures the normative conditions that often precede coercion, including expectations that individuals—especially Black women—should dress or present themselves in sexualized ways because of their racialized gender identity.

To illustrate coercive dynamics shaped by structural vulnerability, research in work-adjacent institutional contexts provides direct evidence. For example, studies of “sex-for-rent” exploitation document explicit sexual demands exchanged for housing stability and protection, with women of color disproportionately targeted—demonstrating how coercion operates through material dependence, constrained choice, and credibility asymmetries (Oliveri, 2015). Legal and intersectional scholarship further underscores how racialized assumptions about credibility and disposability shape the social conditions under which coercion is enacted and resisted, thereby narrowing targets’ practical capacity to refuse or seek institutional redress (Crenshaw, 1989; Hernández, 2018). RSBH advances the literature by theorizing how coercion can be racialized not only through explicit exchange but through the structural and interpretive conditions that make coercion easier to impose and harder to challenge.

Taken together, these behavioral manifestations underscore the central contribution of RSBH: it does not propose new harassment categories but rather explains how race transforms familiar harassment behaviors into mechanisms of racialized gender discipline. Existing frameworks have established that harassment functions to enforce hierarchy and punish norm violations (N. M. Alonso et al., 2025; Berdahl, 2007a; McLaughlin et al., 2012), but they less often specify how sociohistorical racial archetypes shape whose bodies are sexualized, whose boundaries are respected, and whose claims are treated as credible within organizational systems (Buchanan & Ormerod, 2002; Crenshaw, 1989; Settles et al., 2012). By foregrounding meaning, discipline, and power, RSBH clarifies why harassment directed at Black women and Black men is patterned, predictable, and uniquely consequential—even when it appears superficially similar to race-neutral forms of sexual harassment.

### 4.4. Psychological and Behavioral Consequences of RSBH

Harassment has profound and enduring consequences for Black workers’ psychological well-being, identity integrity, and career trajectories. While sexual harassment broadly predicts heightened levels of stress, anxiety, and job dissatisfaction among victims (Willness et al., 2007), the harms of RSBH extend beyond these established outcomes due to the intersectional nature of the violation. Because harassment is rooted in stereotypes that question Black people’s humanity and morality, RSBH inflicts racialized shame—a distressing emotional response tied not just to stigma, but to the degradation of one’s social identity (see Buchanan & Fitzgerald, 2008). Black women who are hypersexualized are often blamed for the behavior they endure, undermining their perception of themselves as deserving of respect and safety (West, 2008; Nelson et al., 2016). Black men subjected to emasculating or criminalizing harassment experience threats to masculinity and belonging, conditions strongly associated with psychological withdrawal, depressive symptoms, and network silence (Hammond, 2012; Hershcovis et al., 2021).

RSBH also shapes perceptions of workplace fairness and trust in organizational institutions. Prior research demonstrates that when harassment is perceived as rooted in racialized bias, targets report stronger beliefs that misconduct is tolerated and that reporting systems lack legitimacy (Berdahl & Moore, 2006; Cortina & Magley, 2009; Fitzgerald et al., 1999). Black employees, especially men, may therefore reasonably anticipate disbelief, retaliation, or minimization if they disclose their experiences. Underreporting is not indicative of resilience or tolerance but rather a rational response to environments that have historically denied the validity of Black suffering or excluded racial minorities from central social ties (see Adikaram & Weerakotuwa, 2025; Collins, 2004; Hershcovis et al., 2021). This environment complicates the ability of racial and ethnic minorities to report harassment, as doing so may lead to ostracism or intensified harassment, particularly for Black men. Their report may trigger increased RSBH, as these victims are seen not just as disrupting the status quo, but as challenging the racial and gender norms that underpin these network structures. This silence compounds psychological harm by allowing harassment to persist unchecked, reinforcing a cycle in which perpetrators are protected while Black workers bear the invisible cost.

Longitudinal evidence suggests that the cumulative nature of intersectional mistreatment erodes Black employees’ occupational health over time. Exposure to racial and gender discrimination predicts higher rates of burnout, job stress, and disengagement, as well as intentions to leave the organization or the broader profession (Buchanan et al., 2007, 2008; Cortina & Berdahl, 2008; Holland & Cortina, 2016; McLaughlin et al., 2017; Raj et al., 2021; Thomas et al., 2008). Beyond psychological and career consequences, repeated exposure to racism and racialized harassment also produces physiological strain. Discrimination has been linked to elevated hypertension, coronary heart disease risk, inflammatory responses, and increased allostatic load—demonstrating that racialized mistreatment becomes biologically embedded over time (Churchwell et al., 2020; Dolezsar et al., 2014; Miller et al., 2021). Black women professionals, including those in academia and healthcare, describe harassment as a force that restricts leadership opportunities and undermines their authority in the eyes of colleagues and supervisors (Holder et al., 2015; Livingston & Pearce, 2009; McLaughlin et al., 2012). Black men, similarly, report that racialized policing of their behavior limits their ability to display competence or ambition without drawing negative attention (Collins, 2004; Wingfield, 2019). In both cases, harassment contributes to career stagnation or employee attrition, reducing diverse representation within high-status domains and reinforcing racialized gender inequality in organizational leadership structures.

Identity-related outcomes are equally consequential. RSBH communicates messages about where Black workers are allowed to belong, how they are permitted to behave, and whose comfort they must prioritize. This persistent identity regulation gives rise to what scholars describe as social identity threat, whereby individuals feel their valued social identity is at risk of being devalued or misrepresented (Steele & Aronson, 1995; Steele et al., 2002). In response, Black workers may engage in emotional regulation strategies such as self-silencing, hypervigilance, or adaptive compliance—each psychologically taxing and associated with diminished authenticity and well-being (Lewis et al., 2016; Purdie-Vaughns & Eibach, 2008; Scott et al., 2023; Settles et al., 2012). Over time, these coping demands lead to cumulative emotional depletion and reduced organizational commitment. Taken together, these consequences illuminate that RSBH is not a private interpersonal problem but a structural barrier to employee success. It not only harms individuals but also diminishes workplaces by driving away high-potential talent, reinforcing occupational segregation, and deepening inequities that organizations often claim to resist. Thus, addressing RSBH is essential not only for legal compliance or reputation management but for the ethical and functional integrity of institutions that rely on high engagement and creativity of their people to thrive.

## 5. Discussion

This paper advances the argument that racialized sex-based harassment (RSBH) represents a distinct mechanism for enforcing racialized gender hierarchies in organizations. Whereas traditional sexual harassment research has conceptualized harassment as motivated by sexual desire or gender status threat (Berdahl, 2007a; Fitzgerald et al., 1995), the present synthesis demonstrates that these frameworks are insufficient without attending to the racialized cultural meanings through which gender is interpreted. We make an important intellectual contribution to the understanding of RSBH by articulating the sociohistorical archetypes that continue to mark Black women and Black men as simultaneously hypervisible and devalued. These archetypes function as interpretive lenses that shape whether harassment is initiated, how it is rationalized, and how organizational members respond to those who experience it. Through this lens, harassment is not simply interpersonal misconduct but a predictable expression of racialized gender hierarchy maintenance.

### 5.1. Theoretical Implications

To be clear, RSBH represents a distinct and patterned mechanism through which race and gender hierarchies are simultaneously enforced in organizational contexts. RSBH captures the convergence of racialized and sexualized domination, reflecting a system of control that uses harassment to discipline Black identity in the workplace and beyond. This framework explains why Black women and men experience higher rates of harassment than their White counterparts and other forms of mistreatment that differ qualitatively in content, attribution, and consequence.

At its core, RSBH functions as an instrument of social dominance maintenance—an enforcement strategy that reasserts racialized and gendered hierarchies when they are perceived to be threatened. When a Black woman demonstrates competence or assertiveness, she may be targeted with sexualized or demeaning remarks meant to restore patriarchal racial order. When a Black man exhibits ambition or vulnerability, he may be subjected to emasculating ridicule or hypersexual suspicion that reestablishes control over his masculinity. These patterns reveal that harassment directed at Black employees is not incidental but a behavioral mechanism that sustains racial-gender hierarchy.

Intersectionality theory (Crenshaw, 1989) clarifies that race and gender cannot be examined as additive categories but intersect to produce unique forms of oppression. Social dominance theory (Sidanius & Pratto, 1999) explains how individuals motivated to preserve group hierarchies engage in discriminatory behavior that reinforces dominance. Integrated threat theory (Stephan & Stephan, 2000) and masculinity threat models (Vandello et al., 2008) illuminate how symbolic and realistic threats to identity and power catalyze harassment behaviors. Indeed, the present work extends harassment theory by demonstrating that racialized gender stereotypes must be understood as mechanisms that activate and justify harassment, rather than as background cultural context. Integrating threat-based models of harassment (Berdahl, 2007a) with research on social dominance orientation and intergroup threat (Sidanius & Pratto, 1999; Stephan & Stephan, 2000) clarifies how RSBH functions to restore power hierarchies when Black women and men are perceived as transgressing racialized gender expectations. Together, these perspectives position RSBH as a form of dominance enforcement that merges structural power with interpersonal bias.

### 5.2. Practical Implications

Failure to recognize RSBH within organizational systems has profound consequences for workplace safety, equity, and performance. Policies must explicitly define RSBH, providing concrete behavioral examples of racialized sexual content and stereotypes that constitute harassment. Training programs should include RSBH-specific case studies to help employees and leaders recognize, name, and disrupt racialized sexual dynamics. Investigators must be trained in controlling-image literacy—the capacity to identify how stereotypes influence perceptions of credibility and harm.

Measurement systems should incorporate RSBH items in climate surveys and reporting tools, allowing organizations to monitor disparities in exposure, reporting, and resolution. Accountability requires auditing disciplinary outcomes to ensure that sanctions are applied consistently and retaliation is prevented. Additionally, restorative and therapeutic interventions must be culturally competent and trauma-informed, recognizing that Black employees may experience unique forms of identity-based harm.

Beyond compliance, addressing RSBH is a matter of organizational integrity and health. Culturally, RSBH constrains authenticity, erodes psychological safety, and limits innovation. Ethically, it represents a violation of dignity that perpetuates inequity and undermines diversity and inclusion initiatives. Scientifically, it demands new frameworks that accurately represent the lived realities of Black workers. Confronting RSBH is thus both a theoretical and moral imperative—central to ensuring equity, belonging, and justice in the modern workplace.

### 5.3. Limitations Future Research Directions

Despite growing recognition of intersectional dynamics in harassment, empirical research remains limited. Future work should examine RSBH as a multilevel process involving individual prejudice, interpersonal interaction, and institutional structures. Methodologically, mixed-method and longitudinal studies can capture how RSBH unfolds over time, how targets navigate identity threats, and how organizational climates either perpetuate or mitigate harm.

Much of the existing research and measurement has focused on Black women’s experiences, and there is limited empirical work capturing the full spectrum of RSBH faced by racially minoritized men, LGBTQ+ individuals, and other marginalized groups (Berdahl, 2007b; Cortina & Areguin, 2021; Settles et al., 2012). Further, as workplace demographics and social dynamics evolve, new forms of intersectional harassment may emerge that are not fully captured by current instruments. Future research should build on Buchanan’s foundation by developing and validating measures for diverse populations, including sociohistorical archetypes as a vehicle to perpetuate harm. Indeed, employing qualitative and mixed-methods approaches and exploring the organizational and societal contexts that shape these experiences remains limited in the harassment research landscape. Thus, advancing a conceptual model of RSBH is both a theoretical imperative and an urgent matter of social and workplace equity.

Experimental research can test whether harassment scenarios containing racialized sexual content elicit lower credibility judgments or weaker disciplinary responses than race-neutral scenarios. Measurement innovation is equally urgent. Existing instruments such as the Sexual Experiences Questionnaire (Fitzgerald et al., 1995) fail to capture the racialized content and cultural imagery that define RSBH. The RSHS (Buchanan, 2016) provides a foundation, but further validation for Black men, LGBTQ+ individuals, those whose racial and gender expression does not conform to dominant norms, and other marginalized groups is necessary.

Integrating biomarkers (e.g., cortisol, blood pressure, C-reactive protein) into RSBH research could connect experiences of harassment with physiological stress responses, advancing a biopsychosocial understanding of harm. Future models should test identity threat, racialized shame, and organizational silence as mediators between RSBH and outcomes such as burnout, turnover, and disengagement.

Finally, the present work is grounded in U.S. racial dynamics, which limits generalizability to cultural contexts where race, ethnicity, and gender are structured differently. The synthesis also focuses primarily on Black cisgender women and men; harassment processes and stereotypes may slightly differ for Black LGBTQ+ workers, nonbinary individuals, and those whose gender presentation diverges from dominant stereotypes. These are not limitations of design but boundaries of scope that point to necessary next steps.

## 6. Conclusions

This research provides a critical advancement in the study of racialized sex-based harassment (RSBH) by framing it as a distinct mechanism of intersectional harm rooted in negative sociohistorical archetypes and dominance enforcement. By integrating threat-based and social dominance theories, the work highlights how harassment functions to regulate Black identity in professional spaces. By addressing the psychological factors underlying RSBH, workplaces and policymakers can develop more effective strategies to protect the most vulnerable individuals in professional, social, and educational settings. Addressing RSBH is not only a theoretical necessity but a moral imperative—one that demands intersectional, culturally responsive strategies to ensure safety, dignity, and equity for Black employees across contexts.

## Figures and Tables

**Table 1:** Sociohistorical archetypes shaping RSBH toward Black men and Black women and their function in the perpetration and experience of racialized sex-based harassment (RSBH).

Archetype	Description	Function in RSBH	Relevant Citations
**Black Women**			
Mammy	Asexual; nurturing caretaker; loyal and servile	Normalizes extraction of care/emotional labor and subordination; denies sexual victimhood	Bogle (2016); Cheeseborough et al. (2020); Collins (2004); Pilgrim (2000b)
Jezebel	Hypersexual; promiscuous, seductive	Rationalizes sexualized remarks, touching, and coercion as inviting or inevitable; denies consent seeking and undermines mobilization of rights	Buchanan (2016); Collins (2004); West (2008)
Sapphire	Hostile; emasculating; aggressive	Justifies exclusion, discipline, and silencing when Black women assert boundaries; delegitimizes grievances as anger	Buchanan and Ormerod (2002); Collins (2004); Lewis et al. (2016)
Strong Black Woman	Emotionally vulnerable; endlessly resilient	Reduces empathy and invalidates organizational support; recasts harm as “she can handle it”	Donovan and West (2015)
Welfare Queen/Gold Digger	Manipulative, financially opportunistic	Frames harassment as justified or rightful punishment	Collins (2004)
**Black Men**			
Uncle Tom/Sambo	Docile, servile, eager to please White authority	Rewards compliance, punishes resistance and leadership	Bogle (2016); Collins (2004)
Mandingo/Black Buck	Hypersexual; dominant; insatiable; predatory; “threat to White woman”	Legitimizes coercive control and heightened identity policing, objectification, dehumanization, and fetishization.	Bogle (2016); Collins (2004)
Brute	Violent, unpredictable, physically threatening	Rationalizes suppression and disciplinary action	Goff et al. (2008); Johnson (2018); Pilgrim (2000a)
Angry Black Man	Hostile, criminal, aggressive	Justifies surveillance, exclusion from leadership, and denial of vulnerability/victimhood	Collins (2004)

## Data Availability

Data sharing is not applicable. No new data were created or analyzed in this study.

## Abbreviations

The following abbreviations are used in this manuscript:

RSBH	Racialized sex-based harassment
RSHS	Racialized Sexual Harassment Scale
